# Acute Tear Secretion Response to Menthol Delivered as a Vapor to the Eye: A Placebo-Controlled, Double-Blind, Randomized Clinical Trial

**DOI:** 10.3390/pharmaceutics18080910

**Published:** 2026-07-24

**Authors:** Reinhold Vieth, Christine Griffiths, Linda M. Rapson

**Affiliations:** 1 Department of Laboratory Medicine and Pathobiology, Faculty of Medicine, University of Toronto, Medical Sciences Building, 5th Floor, King’s College Circle, Toronto, ON M5S 1A8, Canada; 2 Department of Nutritional Sciences, Faculty of Medicine, University of Toronto, Medical Sciences Building, 5th Floor, King’s College Circle, Toronto, ON M5S 1A8, Canada; 3 Stearacl Inc., Toronto, ON M4K 1X4, Canada; 4 Independent Researcher, Toronto, ON M4K 1X3, Canada; 5 Rapson Pain & Acupuncture Clinic, Toronto, ON M4X 1W4, Canada; 6 Department of Family and Community Medicine, University of Toronto, Toronto, ON M5S 1A8, Canada

**Keywords:** menthol, cornea, artificial tears, dry eyes, eye drops, vapor, TRPM8, cold receptors, lacrimal duct

## Abstract

Menthol is an agonist of TRPM8 receptors that activates tear secretion. **Objectives:** The hypothesis was that menthol delivered to the cornea in vapor form could stimulate tear secretion without irritation. **Methods:** This was a randomized, placebo-controlled, double-blind clinical trial of menthol vapor. Thirty generally healthy participants were randomized to the treatment or placebo group. **Results:** Median age was 58 (43, 73 IQR) years, 18 participants (60%) were female; 12 (40%) were male. Tear weight exceeded 5 mg in 2 (13.3%) of the placebo group, versus 13 (86.7%) of the menthol group (*p* = 0.020). Median weight of tears wiped from below the eye in the placebo group was 3 (−1, 8 IQR) mg versus 13 (8, 35 IQR) mg in the menthol group (*p* = 0.002). Nasolacrimal drainage in the placebo group weighed 10 (4,12 IQR) mg versus 56 (22, 173 IQR) mg in the menthol group (*p* < 0.001). The treated right eye felt qualitatively moister than the untreated left eye in 7 (47%) of the placebo group, versus 15 (100%) of the menthol group (*p* = 0.002). No participant reported eye irritation or any adverse effects at the one-week follow-up. **Conclusions:** Menthol delivered to the eye as vapor evoked tear secretion without eye irritation. Menthol vapor warrants further study as a complementary treatment or an alternative to eye drops for dry eye disease.

## 1. Introduction

Menthol is known for its cooling effect when it is applied to the skin. At the eye, menthol-containing eye drops can produce a cooling sensation [1]. However, volunteers receiving menthol-containing eye drops at higher concentrations felt irritation, cold, discomfort, and stinging, without any detectable effect on tear secretion as measured using Schirmer test strips [2].

Tear secretion is the normal response to the cooling from tear evaporation at the cornea, which is mediated by transient receptor potential melastatin-8 (TRPM8) channels. Parra et al. reported that genetic deletion of TRPM8 in mice eliminates cold responsiveness and reduces basal tearing. However, deletion of the TRPM8 gene did not affect pain-mediated irritative tearing. Robbins et al. then showed in mice that applying low-concentration solutions of menthol to the eyes evoked tearing without nociceptive responses. But at concentrations approaching 50 mM, the menthol produced pain responses [3]. In essence, tearing due to a cold sensation is separate and different from tearing due to pain. Whether menthol can be used clinically hinges on its ability to activate cool receptors without causing pain or irritation [4].

The cornea is by far the most sensitive body surface to liquid menthol’s cooling effect [5]. Unlike other body surfaces, the corneal epithelium lacks a stratum corneum, and it is densely innervated. Without the protective barrier, there is a particularly narrow margin of safety at the cornea between menthol’s effects on tearing and pain. There are methods of acutely stimulating reflex tear secretion, but they cause tearing through irritation [6,7,8,9,10,11,12]. Varenicline (TyrvayaTM) is a selective cholinergic agonist that binds to nicotinic acetylcholine receptors in the nasal sinus and causes reflexive tearing and some nasal irritation [11,12]. None of the preceding methods involve TRPM8 receptors.

A curious observation that anyone can make while they are out in winter air (below 5 degrees C) is that even though one may not consciously perceive the coldness in the eyes, tear secretion does go up, as evidenced by the tears still flowing once one returns to an indoor temperature. The same TRPM8 receptors mediate both the skin’s coldness sensation in response to menthol as well as the cornea’s tear-stimulating, dry-sensation response. The difference in conscious perception is the result of how the brain’s somatosensory cortex maps and interprets signals arriving from the skin versus those from the eye. TRPM8 receptor activation at the skin elicits a cooling sensation, while at the cornea, it elicits a tearing response [13,14]. At higher concentrations in the eye, menthol activates pain [2,15].

Clinical evidence involving menthol to stimulate tear secretion has been disappointing. Kovacs et al. applied menthol in paraffin ointment to the cheeks of subjects to release menthol vapors over the eye, but they detected no significant changes in the tearing rate [15]. Arita et al. induced tear secretion using a menthol-impregnated, heated eyelid-warming device [16] but did not continue the investigation. Use of the Schirmer test strip may have confounded the menthol studies of Belmonte et al. and Kovacs et al. [2,15], because placing a strip of filter paper into the conjunctival sac is itself an irritation that can override any subtle effects on tear secretion. With a Schirmer test strip placed under the bottom eyelid, one is no longer measuring the basal tear; one is measuring the irritation response, and its interaction with menthol.

The aim of this randomized clinical trial was to test the hypothesis that menthol delivered to the cornea as vapor arising from topical menthol applied to one cheek might increase tear secretion at the eye above the site of application, without irritating that eye. The thinking was that because the cornea’s TRPM8 receptors naturally respond to the temperature of the surrounding air, which is a gaseous environment, it seemed logical to ask whether menthol delivered to the eye as a vapor in the air might behave differently from its behavior in previous reports delivering menthol directly to the eye in liquid solution [1,2]. If this method of delivering menthol as a vapor improves lubrication at the eye, then it warrants further study as an alternative or complement to the use of artificial tear eye drops for persons afflicted by dry eyes.

## 2. Materials and Methods

### 2.1. Study Design and Participants

This was a single-center, double-blind, vehicle-controlled, phase 2, randomized clinical trial conducted on healthy participants from the community. Participants were invited by word of mouth, and they were fully informed verbally and in print regarding study procedures and risks prior to providing their signed consent to take part. Regardless of whether they completed the study protocol, participants were to be compensated modestly for their participation. The intervention product, as well as the vehicle placebo, and their use for this research protocol had been reviewed by Health Canada, after which Health Canada provided a no-objection letter for use the product for this clinical trial. The clinical trial protocol and consent documentation were reviewed and approved by Veritas Independent Review Board (2025-3748-22021-7; approved on 22 July 2025), and all ethical guidelines were followed throughout the conduct of this work. This clinical trial was registered at ClinicalTrials.gov on 30 July 2025 and given the identifier NCT07109609, at which time all primary and secondary outcomes were specified. The trial was conducted between 25 August 2025 and 10 September 2025. The Consolidated Standards of Reporting Trials (CONSORT) reporting guidelines have been followed for this report.

Persons under the care of a physician for issues related to the eye were excluded prior to the consenting stage; otherwise, generally healthy adults aged 18 years or older were eligible. Prior to conducting the experiment, persons who had consented were excluded if they responded “Yes” to any of the following questions: Are you allergic to menthol or to menthol-containing products like toothpaste or certain cold remedies? Are you wearing contact lenses during the time of testing? Are there tears present prior to applying the menthol? Did you use eye drops during the hour before participation? Do you have broken, irritated, sensitive skin and/or acne at or around the application site? Do you have a history of atopy or allergic diseases on the skin of your cheeks? Do you have Stevens–Johnson syndrome, which is any known skin response to chemical, thermal, or radiation injury to the application area?

### 2.2. Randomization and Masking

Both the active agent and the placebo had been validated by accredited independent laboratories according to formal Health Canada standards. Up to and during the clinical trial, there was never any obvious distinction observed between placebo and treatment doses. While it was later realized that if the top of the open roller bottle was held directly at the nostrils and sniffed, one could detect menthol, such direct smelling of the vial was never part of the protocol.

Preparations of menthol and placebo doses were allocated by a licensed, independent pharmacist who started with a rack of 36 empty, sequentially-numbered vials (5-mL clear-glass vials with stainless-steel roller ball and screw cap). From the first vial number onward, the vials were placed into two racks labeled A and B, based on the drawing of disks from an opaque bag. The bag contained groups of six disks—3 disks marked A and 3 disks marked B. The bag was shaken, and disks were drawn one by one, to allocate each sequentially numbered vial to rack A or rack B. The six disks were returned to the bag to restart the allocation to create a total of six randomly permuted blocks of 6 for the clinical trial. Menthol solution (0.9 mL of 20% *w*/*w* menthol and 80% *w*/*w* medium-chain triglyceride oil) was placed into the vials of rack A, and the placebo vehicle (0.9 mL of 100% medium-chain triglyceride oil) was placed into each of the vials in rack B. The vials were transferred in numerical order into one rack, from which one vial was taken in sequence for each participant. There was no externally discernible difference between vials allocated to the dose groups. Participants, investigators, consenting staff, and the sponsor were all masked to the study treatment. The pharmacist retained the record of dose allocations until after all subjects had completed the trial, and after all the data pertaining to the clinical trial had been entered into a spreadsheet and was ready for analysis. Data entered for analysis was double-checked independently against the original data-recording sheets, prior to unmasking dose allocations. Throughout the study, neither the investigators nor any of the study subjects discerned any difference among the doses.

### 2.3. Intervention and Clinical Assessments

The key outcome measure was the actual weight of secreted tear liquid collected from below the bottom eyelashes, above and away from the point of dose application. Since a runny nose was sometimes noticed during preliminary work, nasal fluid was collected at the end of the 5 min of observation to test the hypothesis that tear fluid might have drained into the nasolacrimal duct. Validation of the nasal collection was tested on 7 consented participants. When they kept their eyes shut during the first 5 min after the menthol application, and then blew the right nostril into tissue, weight increased by 9 mg (1, 18 mg IQR); for the next 5 min, the subjects kept their eyes open, and blew the right nostril into tissue, resulting in a weight gain of 64 mg (52, 218 mg IQR). The difference in tear weight collected when eyes were kept shut with menthol present, versus when eyes were kept open with menthol still present, was significant Wilcoxon Test, *p* = 0.018).

**Figure 1:**
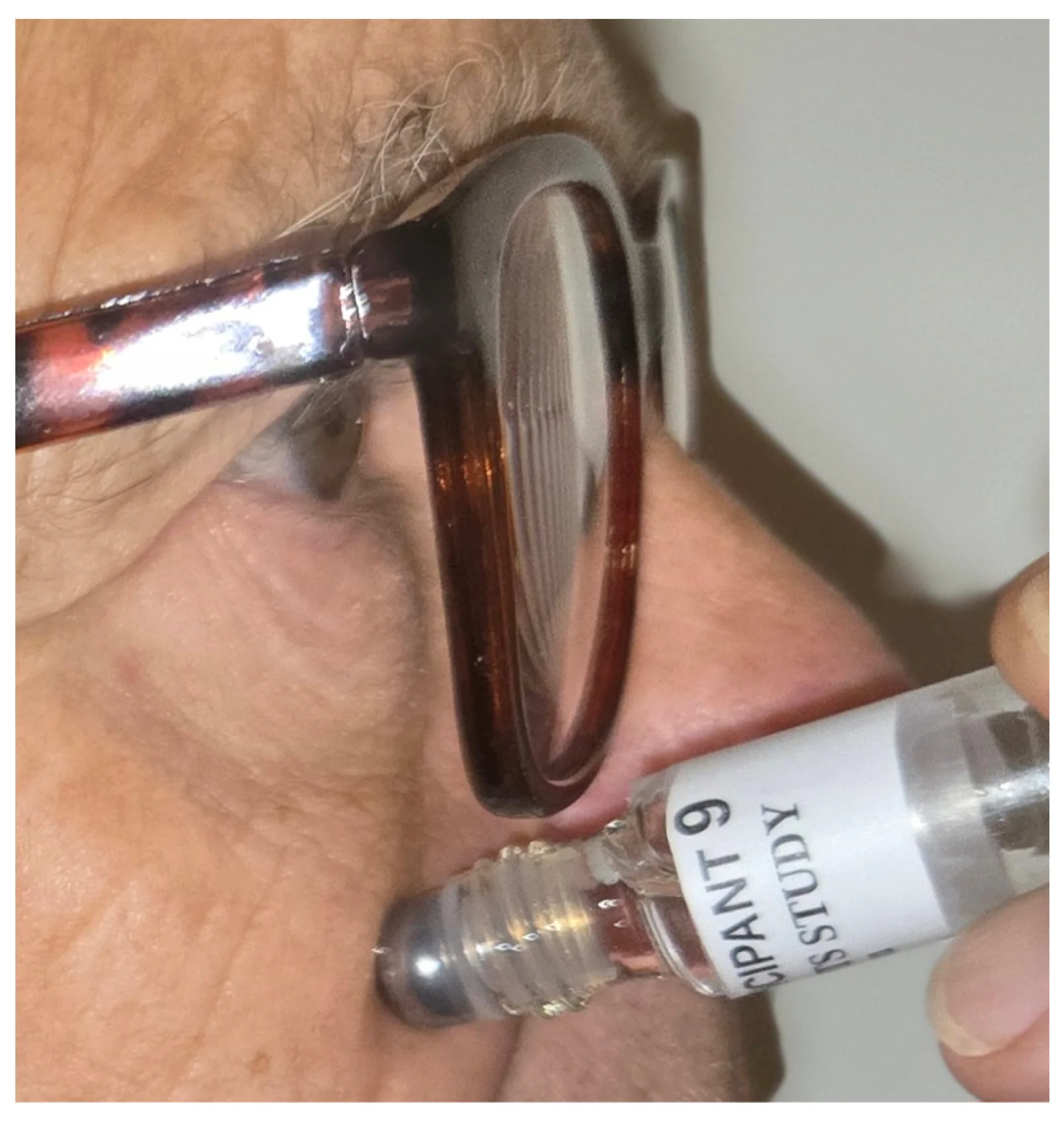
Application site and method for menthol solution or placebo. The illustration shows the rollerball affixed to the vial of solution, shown here at the 2 cm × 2 cm square area of application, superior to and laterally away from the nostrils, below the bottom rim of the eyeglasses.

For the clinical trial, testing was conducted while the subject remained seated indoors in still air. A portable, digital jeweler’s scale, reading to the nearest milligram (SD = 1 mg), was used to measure the absolute weights of the tissue and the 25-mL-volume clear plastic cup that the tissue was placed into for weighing. The portable testing scale made it possible to conduct the testing at locations suitable to participants. Tear fluid weight was based on the weight increases between the weight of the tissue-in-the-cup before and after collecting the sample. To prepare each subject before applying the dose, subjects wrapped a Scotties^®^ facial tissue (Kruger Products L.P., New Westminster, BC, Canada) around a finger and wiped the tissue horizontally across the skin between the bottom eyelashes and the orbital bone of the right eye. If the tissue increased in weight by more than 5 mg, typically due to oils on the skin, the site was wiped again until the weight change was less than 6 milligrams. For preparation, subjects also blew their right nostril into a facial tissue, which was discarded. During the study, all subjects wore glasses, either their own or clear “fake” glasses provided for them. For consistency, the dose solution was applied by the investigator. The dose or placebo was rolled only onto the right cheek by rolling the rollerball around the perimeter and through a square area of about 2 cm × 2 cm at the cheekbone, near the nose, and just outside and below the bottom rim of the glasses that were worn during the 5 min of sitting (Figure 1). The method delivered about a 12 mg solution. This was either placebo oil (medium chain triglyceride, MCT), or the active agent, 2.4 mg menthol as 20% wt/wt in MCT.

A 5-min timer was started, and for the next five minutes, subjects sat in still air with eyes open, but free to blink, read, or speak normally and comfortably. If a tear dripped toward the cheek before the 5 min were up, this was dabbed onto the pre-weighed tissue, and that tissue was also used to wipe at the end of the 5 min. After 5 min, the subject removed the glasses, wrapped the pre-weighed facial tissue around a finger, and wiped again horizontally across the skin between the bottom eyelashes and the orbital bone of the right eye. The weight gain of that tissue was recorded to the milligram, as a measure of tear secreted from the eye (repeated measures of zero-change, untreated tissue, had indicated that the SD of weight change was +/− 1 mg). Next, the subject blew through their right nostril into a pre-weighed tissue. The weight gain of that tissue was recorded to the milligram, as a measure of nasolacrimal drainage during the 5 min, after which subjects were free to wipe the menthol or placebo solution from the cheek; this was done by wiping downward, across the application site, away from the eye.

After the 5-min samples were collected, subjects answered a brief questionnaire (Table 1). One week later, subjects were contacted to inquire about any longer-term adverse effects. The timeline of the preceding steps is presented in Table 1.

### 2.4. Efficacy 2.4 End Points

The binary endpoint used for the power calculation was the presence or absence of tears wiped from below the eyelashes. Based on preliminary testing, if the tissue weight increased by over 5 mg, this was taken as a positive tearing response, and this outcome was used for the statistical power calculation. Other primary endpoints were the absolute weight of tears collected from below the eyelashes of the right eye, and the measure of nasolacrimal drainage based on the absolute weight of the right nostril blow-out into tissue, as well as the subjective sense of a difference in the moisture of the right versus left eyes. Secondary endpoints included a comparison between treated and placebo groups in terms of sense of pain, coolness, or discomfort, and subjective sense of a runny nose. Safety was evaluated immediately after the test procedure by asking about whether there was any pain, and one week later, a follow-up was conducted with each subject by email, inquiring whether there were any residual effects.

### 2.5. Sample Size Calculation

Based on preliminary testing, the sample size was calculated according to the assumption that 20% of the placebo group would exhibit the binary outcome of tear secretion (tissue weight increase exceeding 5 mg), while for the actively treated menthol group, 80% would exhibit tear secretion. The test for proportions using SPSS statistical software Version 31 (IBM) indicated that two groups of 15 subjects would provide a power of 95% to detect the hypothesized odds ratio difference of 16 if true, based on Fisher’s exact test for comparing proportions between two independent groups.

### 2.6. Statistical Analysis

Analyses were performed using SPSS-version-31 statistical software (IBM). The data set included all results for all consented participants. All statistical significance values are presented as 2-tailed. Binary outcomes such as tear secretion onto the cheek and qualitative sense of a runny nose were evaluated using the Fisher exact test, with odds ratio to indicate effect size. Due to the skewed nature of the data, scale measures were compared between groups using the non-parametric, 2-tailed Mann–Whitney U test, with results for each group expressed as median along with the 25th and 75th percentile values (IQR), and the effect size was based on the Hodges–Lehmann estimate for the confidence interval for the median difference between groups. Categorical questionnaire responses relating to degree of severity were compared between groups using the non-parametric, 2-tailed Mann–Whitney U test. No statistical adjustments were made for multiplicity of outcomes.

## 3. Results

### 3.1. Demographic Characteristics and Participant Disposition

**Figure 2:**
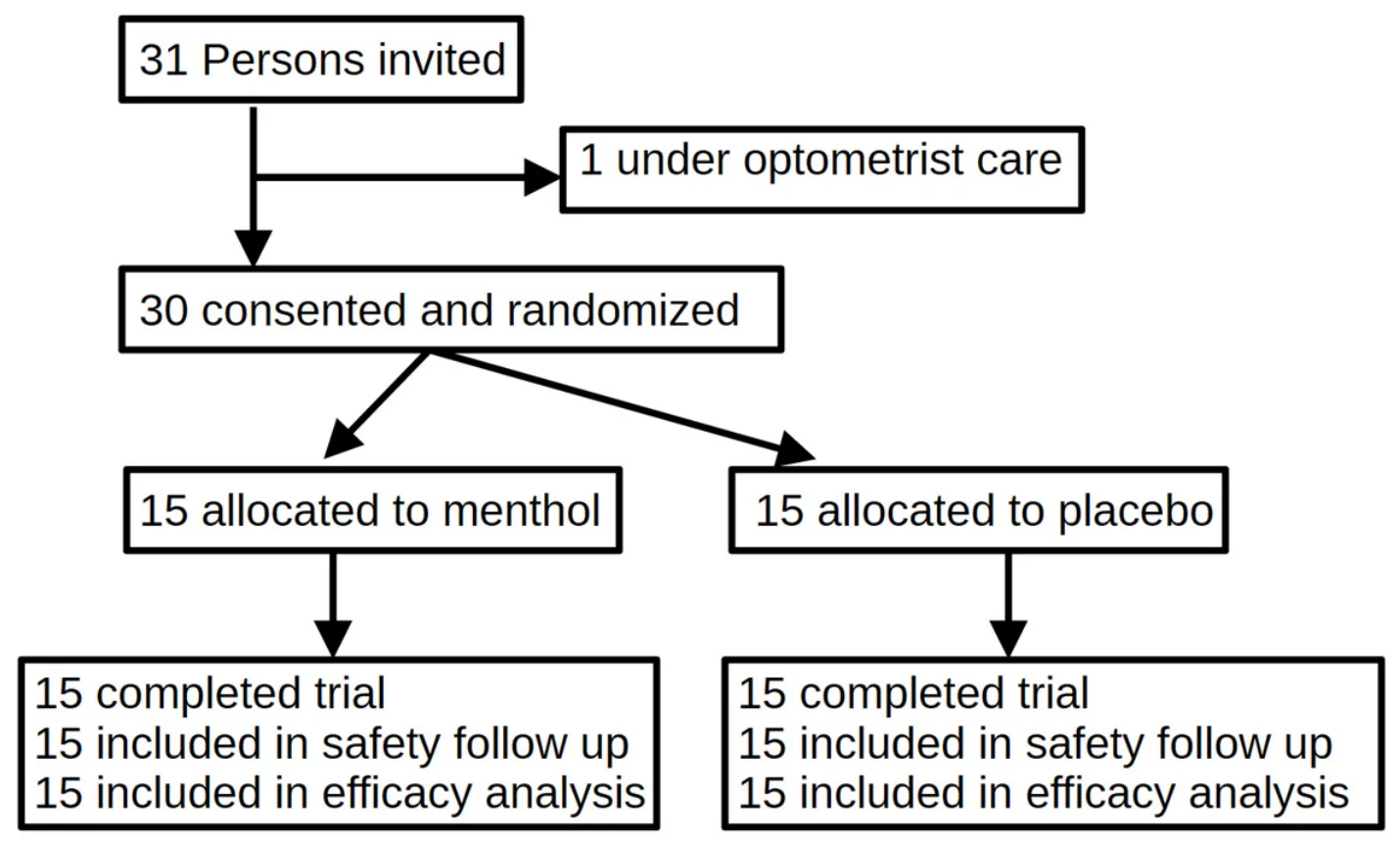
Participant Disposition.

There was no significant difference in gender or age between the groups, as indicated in Table 2. Participant disposition and progress through the study protocol are presented in Figure 2. Safety and tolerability were addressed as part of the study questionnaire, and none of the participants reported feeling pain during or after the 5-min exposure to menthol, neither on the skin of the cheek nor via the vapor. When contacted a week after the procedure, no participant reported any residual effect. No participant withdrew consent, and all participants completed all parts of the experiment.

### 3.2. Primary Outcomes

The binary outcome for tear secretion, based on whether a larger proportion of participants in the menthol group released >5 mg of tears to the bottom eyelid, was significant (*p* = 0.021) (Table 3). Of the 15 subjects in the placebo group, two of them (13.3%) exhibited >5 mg of tears, compared to 13 (86.7%) of the 15 subjects in the menthol-vapor group. In the placebo group, 7 (47%) reported that the right eye felt moister than the left, compared to 15 (100%) in the treated group (*p* = 0.004) (Table 3).

The weight of tears obtained from the skin below the bottom eyelashes was increased by treatment, compared to placebo, by a median of 11.5 mg (4, 2 IQR) (*p* = 0.002). The weight of secreted tears collected from the right sinus as nasolacrimal drainage during the 5 min from application was increased by treatment, compared to placebo, by a median of 46 mg (16, 83 IQR) (*p* < 0.001) (Table 4).

**Figure 3:**
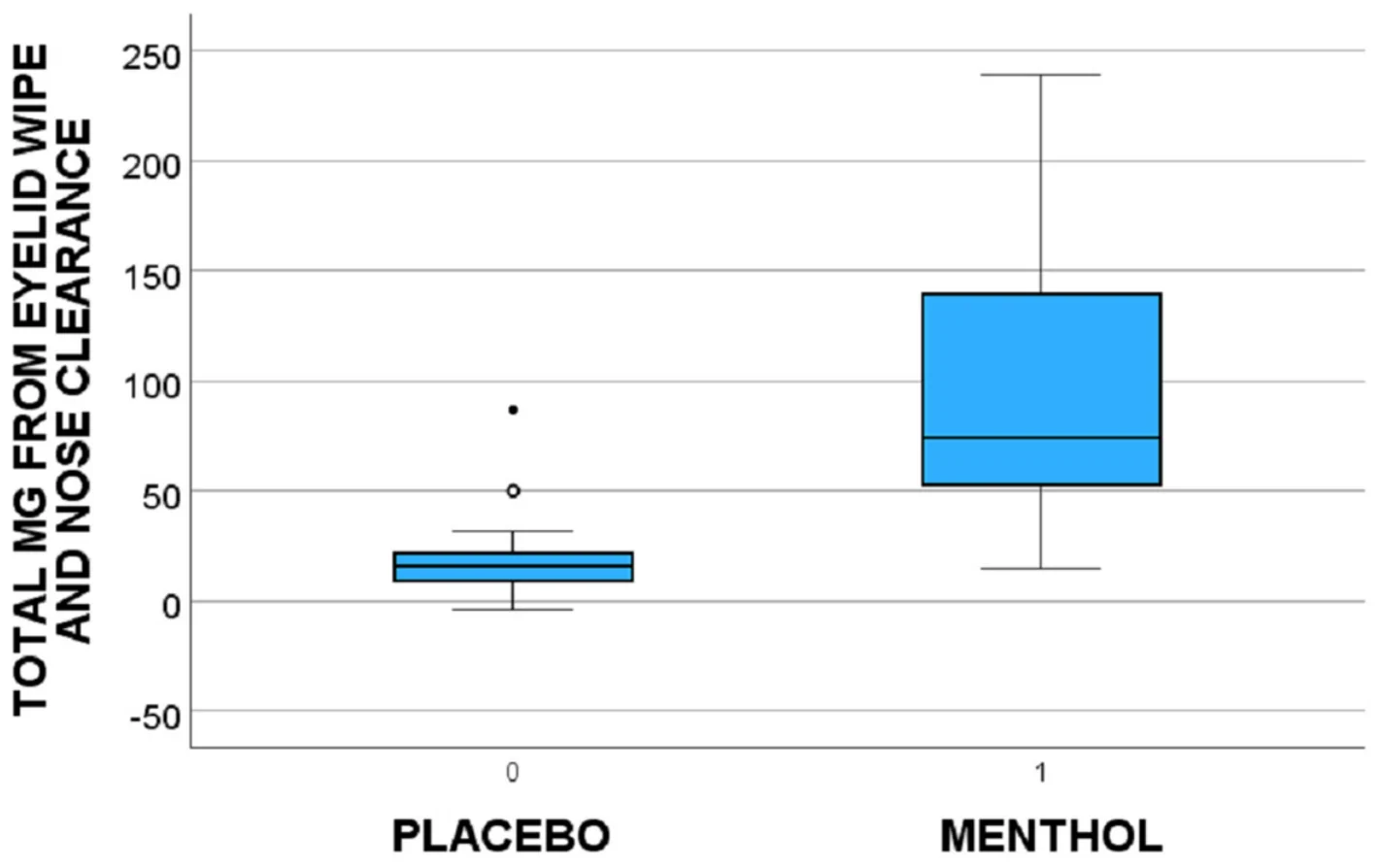
The total mass of fluid collected from wiping the skin below the bottom of the right-side eyelashes, plus the fluid collected from the right nostril after 5 min. N = 15 per group. The boxplots show the median, the 25th, and the 75th percentile values. Dots beyond the whiskers show values classified as outliers by SPSS, i.e., more than 1.5 times the interquartile range beyond the 75th percentile. The difference in medians due to menthol is 58 mg of tear liquid (IQR, 37 and 115 mg).

A composite estimate of the amount of tear fluid that passed from the tear ducts across the right eye shows the sum of tear fluid collected from below the lower eyelashes plus the estimate of nasolacrimal drainage (Figure 3). Compared to placebo, the menthol increased the total tear secretion by 58 (37, 115 95% CI) mg.

### 3.3. Secondary Outcomes

Treatment groups were compared in terms of their subjective responses to questions posed immediately after the tears and nasal fluid weights were recorded. No participant sensed pain at the skin of the cheek where the solution was applied (Table 3). Answers to questions about degree of sensation are presented in Table 5. More subjects in the menthol group reported a sense of coolness on the skin of the cheek (*p* < 0.001). When asked whether they sensed anything at all in the treated right eye, there was no significant difference in eye sensation between the treated and placebo groups (*p* = 0.598. Between groups, there was no significant difference in the small proportion of subjects reporting a subjective sense of a runny nose (Table 3).

**Table 5:** Comparison of ordinal results for cheek coolness and eye sensation between menthol and placebo groups.

	None	Mild	Moderate	Severe	*p*-Value c
Sense of cheek coolness					
Menthol Group (*n*, %, N = 15) c	3 (20.0)	5 (33.3)	6 (40)	1 (6.7)	*p* = 0.002
Placebo Group (*n*, %, N = 15)	12 (80.0)	3 (20.0)	0	0	
Sensation at eye					
Menthol Group (*n*, %, N = 15) c	12 (80)	2 (13.3)	1 (6.7)	0	*p* = 0.598
Placebo Group (*n*, %, N = 15)	14 (93.3)	1 (6.7)	0	0	

Footnotes: c: Mann–Whitney U test.

## 4. Discussion

Menthol vapors arising from 12 mg of a 20% menthol in oil solution applied to the cheek did not irritate the eye, and the vapors were a reliable way to evoke tears in generally healthy subjects. All of the pre-specified primary outcomes were statistically significant. The amount of tears spilling over the bottom eyelashes increased. However, most of the liquid induced by menthol evidently drained down the lacrimal duct. The double-blind, placebo-controlled research study design ensures that the increase due to menthol on nasolacrimal drainage was a true phenomenon related to the treatment. The possibility that the mechanism of action might have been mediated by inhaling some of the menthol vapor is excluded by preliminary testing that showed that keeping the eyes closed while menthol was on the cheek eliminated the effect on tearing or nasal fluid secretion. The difference in the median of the total fluid weights between the groups is a reasonable measure of the sum of nasolacrimal drainage and tear fluid stimulated by menthol. However, rigorous proof that the nasal component of this increase is due to nasolacrimal tear drainage would require using punctal plugs to prevent drainage into the nasal cavity, as well as mucin analysis to isolate secretions originating from the sinuses.

Despite warnings during the consenting process about the possibility of pain with menthol use, none of the participants reported experiencing pain. Furthermore, when study participants were asked the open-ended question, “At your right eye, was there anything uncomfortable feeling?”, 80% of the menthol group and 93% of the placebo group reported that they felt “nothing” (*p* = 0.598). This result is contrary to previous reports using menthol-containing eye drops, where liquid menthol caused cooling or irritation in the eye [1,2]. This also contrasts with the eye irritation that accompanies other ways to stimulate tear secretion [6,7,8,9,10,12]. At the eye, TRPM8-receptor activation by menthol in solution is irritating; however, when the TRPM8-receptor was activated by menthol as a vapor in the air passing over the cornea, it was not irritating, and it stimulated tear secretion.

The use of eyeglasses in this study protocol ensured that the menthol was applied well away from the eye and eliminated the possibility of touching the eye during the monitoring phase. For some participants, the bottom rim of eyeglasses touched the cheek, and this may have impeded the flow of vapor from the application site to the eye. Nonetheless, data for all participants were recorded and analyzed as had been planned.

When the tearing stimulant, Varenicline (TyrvayaTM), is sprayed into the nasal cavity, it stimulates the maxillary branch of the trigeminal nerve, sending a reflex signal to the brain to activate the lacrimal glands [11,12]. In contrast, menthol directly stimulates the TRPM8 cold receptors on the cornea, and the signal travels via the ophthalmic branch of the trigeminal nerve to the trigeminal brainstem sensory nuclear complex [17,18]. The brain interprets this as “surface dryness” or “cooling,” requiring a compensatory response. It warrants further investigation to determine whether the tear film composition, lipid, duration, and volume induced by corneal TRPM8 stimulation differ significantly from the reflex tears generated by the nasal cholinergic response to Tyrvaya.

### 4.1. Future Research Directions

As a diagnostic aid, the rise in reflex tear volume evoked by controlled corneal stimulation by puffs of air has been shown to provide objective information about the tear glands’ secretory capacity in health and disease [9]. Likewise, the response to menthol vapor could provide more specific diagnostic information about the integrity of the TRPM8 receptor signaling system in dry eye disease.

Therapeutically, menthol applied topically, away from the eyes, might be useful as an alternative or complement to the use of eye drops for persons afflicted by dry eyes. Vapor delivery may be safer and more convenient than eye drops, which require sterility and skill to use. Tear fluid provides a composition similar to that of autologous serum (which in itself was designed and intended to mimic the composition of tears). In a meta-analysis, autologous serum eye drops enhance tear secretion, extend tear film break-up time, mitigate corneal epithelial damage, ameliorate OSDI scores, and exhibit greater safety compared to artificial-tear eye drops [19]. In patients with severe dry eye disease, treatment with autologous serum tears significantly reduced dry eye symptoms and showed high patient-satisfaction scores [20]. It makes sense to ask whether TRPM8-stimulated tear secretion with menthol vapor can produce similar results.

Mechanically and quantitatively, tear secretion is different from the way eye drops deliver fluid to the eye. One drop of a commercial eye drop delivers a sudden 30 mg of liquid onto the eye. Most of that is lost from the eye with the first few blinks [21,22]. In contrast to eye drops, menthol vapor caused a much greater flow of tears that lasted for at least 5 min. It is reasonable to ask whether a patient with dry eyes might benefit from the slower release of a larger volume of liquid due to menthol vapor than from a sudden application of a commercial artificial tear solution.

### 4.2. Limitations

There were no eye-care patients involved because the aim was to demonstrate a pharmacological and physiological principle of efficacy without irritation. This clinical trial was limited to a single application of the menthol; therefore, this trial does not provide details about the longer-term use of the menthol-vapor approach in terms of efficacy, safety, and tolerability. There are no objective ocular-surface safety measures reported here, such as conjunctival hyperemia, corneal staining, tear film breakup time, visual symptoms, or slit-lamp examination. Although direct smelling of the vial was not part of the protocol, cheek cooling was more common in the menthol group; that cooling sensation could conceivably have affected the subjective responses, such as eye moisture or sensation of a runny nose. While it is possible that secretion from within the nasal cavity due to allergy or a cold might have affected the estimation of nasolacrimal tear drainage, menthol did not increase the weight of fluid collected unless the eyes were kept open. This finding supports the hypothesis that the nasal fluid increase with menthol was mediated via receptors on the cornea.

## 5. Conclusions

To our knowledge, this is the first randomized, placebo-controlled evidence that delivery of menthol to the eye as a vapor activates tear secretion and improves the sense of moisture without irritation in normal subjects. The total amount of tear fluid induced by menthol vapor was double the amount in a drop of commercial artificial tears. The menthol vapor approach is simple and non-invasive. However, this study does not establish efficacy, tolerability, or safety in dry eye disease. The results should motivate further research to determine how and whether patients with dry eye disease might benefit, either acutely or in the longer term, from menthol-vapor therapy.

## Figures and Tables

**Table 1:** Study design.

Day and Time	Assessment
Day 1, at 5 min after application	Tear weight binary (>6 mg)
Day 1, at 5 min after application	Tear weight mg
Day 1, at 5 min after application	Nose blowout mg
Day 1, at 5–10 min after application	Subjective eye moisture difference
Day 1, at 5–10 min after application	Sensation at eye
Day 1, at 5–10 min after application	Skin pain or skin cooling score
Day 1, at 5–10 min after application	Sense of runny nose (yes/no)
One week later, follow up	Follow-up regarding adverse effects

**Table 2:** Demographic and baseline characteristics.

	Menthol	Placebo	*p*-Value
Characteristics	(N = 15)	(N = 15)	
Age (median, IQR)	64 (45, 74)	51 (42, 73)	>0.05 c
Sex			
Female (*n* (%))	8 (53.3)	10 (66.7)	>0.05 b
Male (*n* (%))	7 (46.7)	5 (33.3)	>0.05 b

Footnotes: N: number of subjects in the group; *n*: number of observations in the category; b: Fisher’s exact test, 2-tailed, between groups; c: Mann–Whitney U test.

**Table 3:** Comparison of binary response outcomes between menthol and placebo groups.

	Menthol	Placebo	*p*-Value b	Confidence Interval
	N = 15	N = 15		OR (IQR)
Tears detected (*n*, (%)) a	13 (86.7)	2 (13.3)	*p* = 0.021	9.75 (1.59, 9.70)
Which eye felt more moist?				
Left eye (*n* (%))	0	2 (13)		
No difference (*n* (%))	0	6 (40)		
Right eye (*n* (%))	15 (100)	7 (47)	*p* = 0.002	2.14 (1.25, 3.68)
Was there a sense of pain (*n*, %)	0	0	NS	
Sense of a runny nose (*n*, %)	4 (26.7)	0 (0)	*p* = 0.100	

Footnotes: a: Binary, yes if tear weight > 5 mg; b: Fisher’s exact test, 2-tailed, between groups; *n*: number of observations in the category; N: number of subjects in the group.

**Table 4:** Comparisons of tear and nasal fluid weights between menthol and placebo groups.

	Menthol N = 15	Placebo N = 15	*p*-Value	Confidence Interval for mg Difference
Tear Weight mg (median, IQR)	13 (8, 35)	3 (−1, 8)	*p* = 0.002 c	11.5 (4, 27) d
NL Drainage mg (median, IQR)	56 (22, 173)	10 (4, 12)	*p* < 0.001 c	46 (16, 83) d

Footnotes: c: Mann–Whitney U test. d: Independent samples Hodges–Lehman Median confidence interval for the difference between median values.

## Data Availability

Deidentified results and data for this trial are available a an Excel file, submitted as Supplementary Files to this article.

## Supplementary Materials

The following supporting information can be downloaded at: https://www.mdpi.com/article/10.3390/pharmaceutics18080910/s1. The file conatains the data as analyzed by the SPSS statistical software, with headers atop each data column named in a manner that explains the data coding.

## Abbreviations

The following abbreviations are used in this manuscript:

CONSORT	Consolidated Standards of Reporting Trials
IQR	Interquartile range
MCT	Medium chain triglyceride oil
NL	Nasolacrimal
OR	Odds ratio
TRPM8	Transient receptor potential melastatin-8 (TRPM8)
